# E-cadherin Plays a Role in Hepatitis B Virus Entry Through Affecting Glycosylated Sodium-Taurocholate Cotransporting Polypeptide Distribution

**DOI:** 10.3389/fcimb.2020.00074

**Published:** 2020-02-27

**Authors:** Qin Hu, Feifei Zhang, Liang Duan, Bo Wang, Yuanyuan Ye, Pu Li, Dandan Li, Shengjun Yang, Lan Zhou, Weixian Chen

**Affiliations:** ^1^Department of Laboratory Medicine, The Second Affiliated Hospital of Chongqing Medical University, Chongqing, China; ^2^Key Laboratory of Laboratory Medical Diagnostics of Ministry of Education, Chongqing Medical University, Chongqing, China

**Keywords:** hepatitis B virus (HBV), virus entry, HBV co-receptor, E-cadherin, NTCP

## Abstract

Hepatitis B virus (HBV) infection is a major cause of chronic liver disease and hepatocellular carcinoma. Current antiviral therapy does not effectively eradicate HBV and further investigations into the mechanisms of viral infection are needed to enable the development of new therapeutic agents. The sodium-taurocholate cotransporting polypeptide (NTCP) has been identified as a functional receptor for HBV entry in liver cells. However, the NTCP receptor is not sufficient for entry and other membrane proteins contribute to modulate HBV entry. This study seeks to understand how the NTCP functions in HBV entry. Herein we show that knockdown of the cell-cell adhesion molecule, E-cadherin significantly reduced infection by HBV particles and entry by HBV pseudoparticles in infected liver cells and cell lines. The glycosylated NTCP localizes to the plasma membrane through interaction with E- cadherin, which increases interaction with the preS1 portion of the Large HBV surface antigen. Our study contributes novel insights that advance knowledge of HBV infection at the level of host cell binding and viral entry.

## Introduction

Hepatitis B virus (HBV), a member of the *Hepadnaviridae* family, is a small, enveloped DNA virus (Glebe and Bremer, 2013) that infects human liver parenchymal cells. Although the widespread use of vaccines has greatly reduced the rate of infection, HBV continues to pose a serious threat to global health, affecting more than 350 million individuals worldwide, all of whom are at increased risk of developing liver cirrhosis and hepatocellular carcinoma (HCC) (Trepo et al., 2014). Current therapeutic regimens that employ direct-acting antivirals, with or without ribavirin, have significantly increased the prevalence of escape mutants, caused significant adverse effects while eliciting a low curative rate in HBV patients (Gish et al., 2012).

The lack of effective therapeutic options for HBV is partially due to our incomplete understanding of the HBV life cycle, including the stages during which the virus enters host cells, undergoes DNA replication, and assembles and releases virions from the cells. Productive HBV infection occurs following viral entry into a host cell, which is initiated through the binding of preS1 the domain of HBV large envelope protein (LHBs) with high affinity HBV receptors on hepatocytes (Le Duff et al., 2009; Glebe and Urban, 2017). The sodium-taurocholate cotransporting polypeptide (NTCP), a bile acid transporter expressed at the basolateral membrane of human hepatocytes, has been identified as a functional receptor for HBV (Yan et al., 2012). Exogenous expression of NTCP in human hepatoma cell lines, such as HepG2 or Huh7, confers susceptibility to HBV infection, and thus, constitute effective cell culture models for examining HBV entry (Yan et al., 2012; Ni et al., 2014). However, the overexpression of NTCP in human or extrahepatic human cell lines, such as HeLa cells, or in mouse hepatocytes, is insufficient for productive HBV infection in these cells, suggesting that additional molecules are also required for efficient HBV infection (Tong and Li, 2014).

The overexpression of the hepatitis B surface antigen binding (SBP) protein in HepG2 cells (HepG2-SBP) induced susceptibility to HBV infection. SBP was shown to interact directly with HBV envelope proteins (Sun et al., 2018). Moreover, heparin sulfate proteoglycans can function to bind HBV and bring the virus to the proximity of the NTCP receptor (Schulze et al., 2007). Verrier et al. (2016) also reported that Glypican 5 attaches to the surface of HBV particles prior to NTCP binding, thereby assisting in host cell entry. These studies indicated that other molecules in addition to NTCP have important roles in efficient and productive HBV infection.

Cadherin adhesion molecules are core components in adherens junctions, located on the basement membrane aspect of polarized epithelial cells. E-cadherin is a calcium-dependent adhesion integrin that is abundant in epithelial tissues and plays an important role in cell-cell adhesion complexes including desmosomes and adherens junctions (Fonseca et al., 2018). E-cadherin play a role in pathogen infection (Bonazzi and Cossart, 2011). Herein, we investigated the role and mechanism for E-cadherin to modulate HBV infection.

## Materials and Methods

### Cell Lines

HepG2-NTCP, HepAD38 (in which HBV replication can be regulated by tetracycline), and Huh-7cells, were provided by Professor Juan Chen of the Chongqing Medical University, Chongqing, China. HepG2-NTCP and HepAD38 cells were maintained in Dulbecco's Modified Eagle Medium (DMEM, Hyclone, Logan, UT, USA) supplemented with 10% fetal bovine serum (FBS, Gibco, Franklin Lakes, NJ, USA) and 400 μg/mL G418. HepG2-NTCP cells were cultured with collagen pretreatment. Huh-7 cells were maintained in DMEM containing 10% FBS. HepaRG cells were purchased from Beijing Beina Science and Technology (Beijing, China) and were cultured in William's E Medium (Sigma-Aldrich, St. Louis, MO, USA) with 10% FBS, 2 mM L-glutamine, 5 μg/mL insulin (Sigma-Aldrich), and 50 μM hydrocortisone hemisuccinate (Sigma-Aldrich). Primary human hepatocytes (PHH) were obtained from ScienCell Research Laboratories (Carlsbad, CA, USA) and were maintained in Hepatocyte Medium (HM, Catalog no. 5210, ScienCell Research Laboratories). All cells were cultured in a humidified incubator at 37°C with 5% CO_2_.

### Plasmids and Gene Products

The pNL4-3.Luc.R-E- reporter plasmid was obtained through the NIH AIDS Reagent Program. The plasmid of pNL4-3.Luc.R-E- was constructed by rendering this clone Env- and Vpr- with frameshift and inserting the luciferase into the pNL4-3 nef gene, the HIV-1 proviral clone (Connor et al., 1995; He et al., 1995). The pcDNA3.1-E-cadherin plasmid was obtained from Addgene (Cambridge, MA, USA). Partial cDNA for human E-cadherin subcloned into the pcDNA3.1 to generate the pcDNA3.1-Ecad-Δ 35 and then the 425-bp fragment encoding the missing COOH-terminal residues and generated by PCR was sub-cloned into pcDNA3.1-Ecad-Δ 35 to generate full-length pcDNA3.1-E-cadherin plasmid (Gottardi et al., 2001). The pcDNA3.1-HBV1.3 (replication-competent 1.3-fold overlength HBV) and the pcDNA3.1 (vector) plasmids were provided by Professor Juan Chen of Chongqing Medical University (Chongqing, China).To study the effect of E-cadherin on HBV binding to HepG2-NTCP cells, the FITC- and Biotin-labeled preS1 peptides encoding the stable region of preS1 (2-47aa) was synthesized by Zhejiang Baitai Biological Company (Zhejiang, China). The sequence used to produce the FITC- and Biotin-labeled preS1 peptides was: Myr-GTNLSVPNPLGFFPDHQLDPAFGANSNNPDWDFNPNKDHWEANQVK (Yan et al., 2012). The second amino acid of the preS1 peptides was myristoylated.

### Protein Extraction and Western Blotting Analysis

Total protein was extracted from cells using a Protein Extraction Kit (KaiJi, Tianjin, China) according to the manufacturer's instructions. The concentration of protein was quantified via bicinchoninic acid (BCA) protein assay (Beyotime, Shanghai, China) according to the manufacturer's instructions. Equal amounts of total protein from each sample (50 μg) were separated using sodium dodecyl sulfate-polyacrylamide gel electrophoresis (SDS-PAGE) and transferred to a nitrocellulose membrane. The membranes were incubated with rabbit anti-E-cadherin (1:1,000; Cell Signaling Technology, Boston, MA, USA), rabbit anti-HBc (1:1,000; Dako, Glostrup, Denmark), rabbit anti-NTCP (1:1,000; Sigma-Aldrich), rabbit anti-Na^+^-K^+^-ATPase (1:1,000; Abgent, San Diego, CA, USA), and anti-actin (1:1;000; Boster, Wuhan, China) primary antibodies at 4°C overnight, and subsequently incubated with horseradish peroxidase (HRP)-conjugated secondary antibodies (1:10,000; Boster) at 37°C for 1 h. Blots were developed using an enhanced chemiluminescence reagent (Pierce, Rockford, IL, USA) according to the manufacturer's instructions. The gray value of blots was calculated by the Image Lab.

### Preparation of Cell Membrane Fractions

Cell membrane proteins were isolated using Minute^TM^ Plasma Membrane Protein Isolation and Cell Fractionation Kit (ScienCell Research Laboratories) according to the manufacturer's instructions. Protein concentrations in the extraction samples were quantified using BCA protein assays (Beyotime) according to the manufacturer's instructions.

### Immunofluorescence Assay

Coverslips containing cells were washed with phosphate buffered saline (PBS) twice, fixed with 4% paraformaldehyde at 37°C for 10 min, blocked with goat serum at 37°C for 1 h, and incubated with primary antibodies, such as rabbit anti-HBs (1:100; Abcam, Catalog no. ab68520, Cambridge, UK) and rabbit anti-NTCP (1:100; Sigma-Aldrich) at 4°C overnight. The coverslips were then incubated with secondary antibodies (FITC-anti-rabbit IgG, 1:100; Boster) at 37°C for 1 h, and stained with 4′,6-diamidino-2-phenylindole (DAPI) at 37°C for 8 min. Immunofluorescence analysis were performed using a DFC5000 camera (Leica, Wetzlar, Germany) attached to an FV500 Confocal Laser Scanning microscope (Olympus, Tokyo, Japan). The virus infection rate of the cells is the percentage of cells expressing green fluorescence calculated by the ImageJ-win 64.

### Analysis of Messenger RNA Expression by Quantitative Real-Time Polymerase Chain Reaction

Total RNA was extracted from cells using TRIzol reagent (Invitrogen, Carlsbad, CA, USA) according to the manufacturer's instructions. Reverse-transcription of total RNA was performed using PrimeScript RT Reagent Kit with gDNA Eraser (Takara Shiga, Japan). Quantitative real-time polymerase chain reaction (qRT-PCR) of the HBV 3.5 kb mRNA was performed using SYBR Green Master Mix (Roche, Basel, Switzerland). The primers for the HBV 3.5 kb were: forward: 5′-GCCTTAGAGTCTCCTGAGCA-3′ and reverse: 5′-GAGGGAGTTCTTCTTCTAGG-3′. The primers for the E-cadherin were: forward: 5′-CCCACCACGTACAAGGGTCAGGT-3′ and reverse: 5′-ACGCTGGGGTATTGGGGGCA-3′. The primers for the NTCP were: forward: 5′-CCACAACGCGTCTGCCCCAT-3′ and reverse: 5′-TGCAGCCCAGCGAGAGCATG-3′.The primers for β-actin were: forward: 5′- TCCCTGGAGAAGAGCTACGA-3′ and reverse: 5′-AGCACTGTGTTGGCGTACAG−3′. The qRT-PCR was run on a Bio-Rad with the following protocol: 2 min at 95°C to pre-denaturation, followed by 40 cycles of denaturation at 95°C for 20 s, annealing at 60°C for 20 s, extension at 72°C for 20 s. All values were normalized to β-actin expression and were calculated using the 2^ΔΔCT^ method.

### siRNA Transfections

The concentration of siRNAs is 20 pmol/μl. 100 pmol of targeting small interfering or non-targeting siRNAs were transfected into HepG2-NTCP, HepaRG or PHH cell lines planted into 6-well plate using siRNA-mate (Genephama, Shanghai, China) according to the manufacturer's protocol. Further treatments were typically performed 72 h after siRNA transfection began, when gene silencing was determined to reach peak efficiency. The sequence used for siRNA targeting E-cadherin was: 5′-CAGACAAAGACCAGGACUA-3′. The sequence of the siRNA targeting NTCP was: 5′-CAGUUCUCUCUGCCAUCAA-3′. The sequence of the negative control siRNA was: 5′- UUCUCCGAACGUGUCACGUdTdT-3′.

### HBV Particle Production and Infection

HBV particles were enriched from the supernatant of HepAD38 cells using 8% PEG8000 (Sigma-Aldrich) (Ladner et al., 1997). HepG2-NTCP, HepaRG and PHH cells were seeded in 24-well plates and maintained in William's E Medium supplemented with 10% FBS, 2 mM L-glutamine, and 2% DMSO for 1 d at the time of HBV infection. HBV infection was assessed at 2 d by qRT-PCR quantification of HBV 3.5 kb RNA, and at 3 d via western blotting analysis of HBV core protein (1:1,000; Dako), and immunofluorescence assay of HBsAg, using the rabbit anti-HBsAg antibody (1:100; Abcam).

HBV particles from adult chronic HBV patients were obtained and used for infection as previously described (Sun et al., 2018). Briefly, HepG2-NTCP and PHH cells were seeded at a density of 5 × 10^4^ per well into 24-well plates. On the following day, cells were incubated with the HBV-positive serum (>5 × 10^7^ copies/mL) diluted in DMEM at a multiplicity of infection (MOI) of 100. Infected cells were maintained in DMEM or HM with 2% FBS. The HBV 3.5 kb RNA, HBc and HBsAg were assessed at 4d post-infection.

### Packaging and Infection of HBV Pseudotyped Particles

HBV pseudotyped particles (HBVpps) can infect liver cells but cannot replicate in human hepatocytes. Infection and packaging of HBVpps were achieved as previously described (Meredith et al., 2016). Infectious virus can be produced by co-transfection with HBV membrane protein expression vector. Therefore, HBVpps were produced by co-transfecting pNL4-3.Luc.R-E- and pcDNA3.1-HBV1.3 plasmids into Huh7 cells using Lipofectamine 2000 Transfection Reagent (Invitrogen). The supernatants containing HBVpps were harvested 72 h after transfection and centrifuged at 1,500 × *g* for 15 min to remove debris. The pseudotyped particles were then added to 96-well microplates, which had been seeded with target cells at a density of 5 × 10^3^ on the previous day, at a volume of 100 μL/well. The medium was supplemented with polybrene (Santa Cruz, CA, USA) at a final concentration of 4 μg/mL. Twenty-four hours after infection, 100 μL of fresh medium was changed per well. Luciferase assays were performed after 72 h using a GloMaxTM 96 Microplate Luminometer (Promega, Madison, WI, USA).

### Co-immunoprecipitation Assay

Co-immunoprecipitation assay was performed as previously described (Sun et al., 2018). Cells were lysed in immunoprecipitation buffer supplemented with complete protease inhibitor. After lysing with ultrasound, lysates were centrifuged to remove insoluble components. Lysates were then incubated overnight with rabbit anti-E-cadherin (Cell Signaling Technology) or rabbit IgG primary antibodies at 4°C. Protein G beads (Beyotime) were then added to the lysates. The precipitated proteins were measured via western blot analysis as described above.

### Pull-Down Assay

Magnetic beads (ThermoFisher Scientific, Waltham, MA, USA) were incubated with biotin-labeled preS1 antibodies at 37°C for 1 h according to the manufacturer's instructions. The beads were washed thrice with TBST, incubated with 500 μL HepG2-NTCP cell lysate at 4°C overnight, and washed a further three times. Thirty microliters of SDS-PAGE reducing sample buffer was then added to each tube and heated at 100°C in a heating block for 5 min. The precipitated proteins were quantified via western blotting analysis.

### CCK-8 Cell Proliferation Assay

HepG2-NTCP and HepaRG cells were seeded in 96-well plates (5 × 10^3^ cells in 100 μl per well). At different time points, 10 μl of cell counting kit-8 (CCK-8) solution (Dojindo, Shanghai, China) was added and incubated for 2 h. The value of OD was measured at 450 nm in a microplate reader (ThermoFisher Scientific, Waltham, MA, USA).

### Detection of HBsAg Expression With ELISA

HBsAg in culture samples collected from the infected cells at 4 days was detected with ELISA kits (LiZhu, Guangdong, China) according to the manufacturer's instructions. The value of cut off is equal to the negative value plus 0.09. The value of S/CO is the ratio of OD to the value of cut off.

### HBV Pre-S1 Binding and Internalization Assay

The experiment was performed as the previous study (Sun et al., 2018). Five microgram of FITC-preS1 was added into the HepG2-NTCP or PHH cells in 96-well plate. The cells were incubated at 4°C for 2 h and then washed with PBS for three times. We determined the binding of pre-S1 by observing cells with linear or dot-like fluorescence on the cell membrane with fluorescence microscope. Besides, incubate it at 37°C for 2 h and then wash it with PBS for three times. We determined the internalization of pre-S1 by observing the green fluorescence inside the cell. We counted the number of cells in white light and then counted the number of cells that express fluorescence in the cell membrane or inside the cell.

### Ethics Statement

We collected fresh serum from adult chronic HBV patients. The study was approved by the Ethics Committee of the Second Affiliated Hospital of Chongqing Medical University. This research was conducted according to the relevant guidelines, and all participants gave written informed consent before the experiment.

### Statistical Analyses

Statistical Package for the Social Sciences (SPSS) 16.0 was used to perform unpaired Student's *t*-tests. Results with *p* < 0.05 were considered statistically significant. Each experiment was repeated a minimum of three times.

## Results

### Downregulation of E-cadherin Reduces Infection Efficacy of HBV Particles

**Figure 1:**
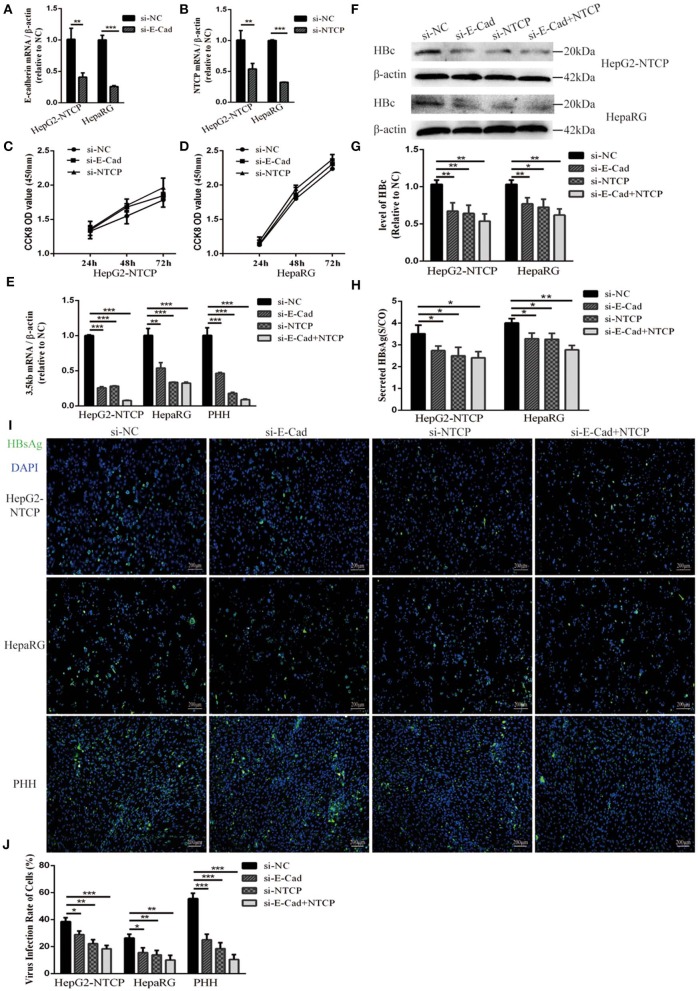
Downregulation of E-cadherin reduces infection efficacy of HBV particles enriched from supernatants of HepAD38 cells. **(A)** E-cadherin expression was silenced in HepG2-NTCP and HepaRG cells by siRNAs. **(B)** NTCP expression was silenced in HepG2-NTCP and HepaRG cells by siRNAs. Silencing efficacy was assessed for E-cadherin and NTCP by qRT-PCR 2 days post-transfection. The value of si-NC group was normalized to 1. **(C,D)** CCK8 assay showed proliferation of HepG2-NTCP and HepaRG cells transfected with si-NC, si-E-cadherin, and si-NTCP. There was no significant difference between the groups. **(E)** Total RNA was extracted 2 days post-infection and HBV infection was assessed by qRT-PCR quantification of HBV 3.5 kb mRNA normalized to β-actin mRNA. The value of si-NC group was also normalized to 1. **(F)** Total protein was extracted 3 days post-infection and expression of HBV core (HBc) was assessed by western blot analysis in HepG2-NTCP and HepaRG cells. **(G)** The densitometric ratios were normalized to the β-actin and then compared to the controls. **(H)** HBsAg in culture collected from the infected cells at 4 days was detected with ELISA kits in HepG2-NTCP and HepaRG cells. The value of S/CO is the ratio of OD to the value of cut off. **(I)** HBsAg expression was assessed by immunofluorescence 3 days post-infection in HepG2-NTCP, HepaRG and PHH cells treated with si-NC, si-E-cadherin, si-NTCP or si-E-cadherin and si-NTCP together. **(J)** The virus infection rate of the cells is the percentage of cells expressing green fluorescence calculated by the ImageJ-win 64. Representative data is shown from triplicate experiments. *p < 0.05, **p < 0.01, ***p < 0.001; error bars: standard deviation (SD).

HepG2-NTCP cells that stably express a recombinant NTCP receptor, PHH from human donors, and HepaRG cells that differentiated into hepatocytes can be infected by HBV in culture (Schulze et al., 2012; Ren et al., 2018). We used these cells to elucidate the role of E-cadherin in modulating HBV infection. HepG2-NTCP, HepaRG, and PHH cells were transfected with 20 pmol of siRNA-NC, siRNA-E-cadherin, siRNA-NTCP or a combination of siRNA-E-cadherin and siRNA-NTCP before infection with 1 × 10^3^ genome equivalents/cell of HBV produced from HepAD38 cells in 24-well plate. The mRNA level of E-cadherin and NTCP were downregulated about or more than 50% following siRNA treatment in HepG2-NTCP and HepaRG cell lines (Figures 1A,B). Transfection with siRNA had no effect on viability in HepG2-NTCP and HepaRG cells (Figures 1C,D). Moreover, silencing of E-cadherin and NTCP significantly reduced the level of HBV 3.5 kb mRNA in HepG2-NTCP, HepaRG, and PHH cell lines (Figure 1E); while silencing of both E-cadherin and NTCP in HepG2-NTCP and PHH cells served to further reduce the level of HBV 3.5 kb mRNA compared to that observed by either E-cadherin or NTCP separately. Western blot, ELISA and immunofluorescence analysis determined that downregulation of E-cadherin or NTCP independently, or together, reduced the levels of HBV core protein and HBsAg (Figures 1E–J). These results suggest that reducing E-cadherin levels reduced productive HBV infection.

### Silencing of E-cadherin Inhibits Infection With HBV Particles Isolated From the Serum of an HBV Carrier

**Figure 2:**
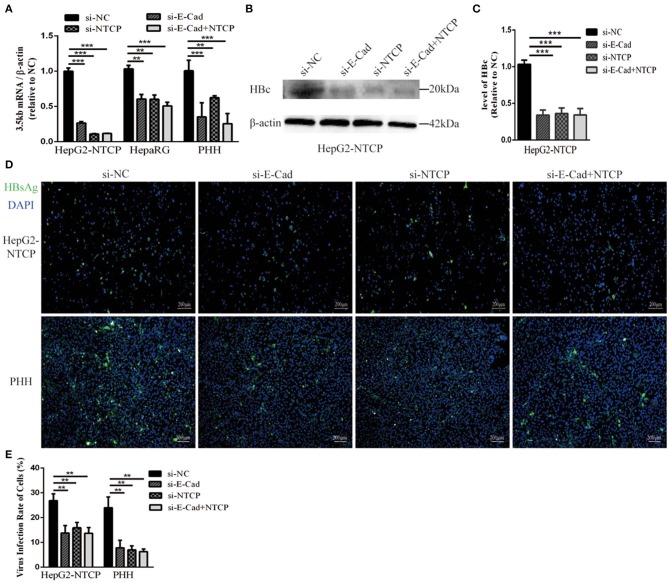
Downregulation of E-cadherin inhibits infection of HBV particles from the serum of an HBV carrier. HepG2-NTCP, HepaRG and PHH cells were incubated with the HBV-positive serum diluted in DMEM at an MOI of 100 after 3 days post-transfection with si-NC, si-E-cadherin, si-NTCP, or si-E-cadherin and si-NTCP together. **(A)** Total RNA was extracted 4 days post-infection and HBV infection was assessed via qRT-PCR quantification of HBV 3.5 kb mRNA normalized to β-actin mRNA. The value of si-NC group was also normalized to 1. **(B)** Total protein was extracted 4 days post-infection and HBV core (HBc) expression was assessed by western blot analysis in HepG2-NTCP cells. **(C)** The densitometric ratios were normalized to the β-actin and then compared to the controls. **(D)** HBsAg expression was assessed by immunofluorescence 4 days post-infection in Hep2-NTCP and PHH cells. **(E)** The virus infection rate of the cells is the percentage of cells expressing green fluorescence calculated by the ImageJ-win 64. Representative data is shown from triplicate experiments. **p < 0.01; ***p < 0.001; error bars: standard deviation (SD).

To further elucidate the relationship between E-cadherin and HBV infection, HepG2-NTCP and PHH cells were infected with HBV particles obtained from a chronic HBV patient. Infection were detected after 4 days. Silencing of E-cadherin or NTCP alone or at the same time, the levels of HBV 3.5 kb mRNA were downregulated about or more than 50% in HepG2-NTCP and PHH cells (Figure 2A). Moreover, silencing of E-cadherin also significantly inhibited the level of HBV core and HBsAg proteins (Figures 2B–E). These results demonstrate that reducing E-cadherin inhibited infection by HBV isolated from an HBV patient.

### Overexpression of E-cadherin Promotes HBV Particles Infection

**Figure 3:**
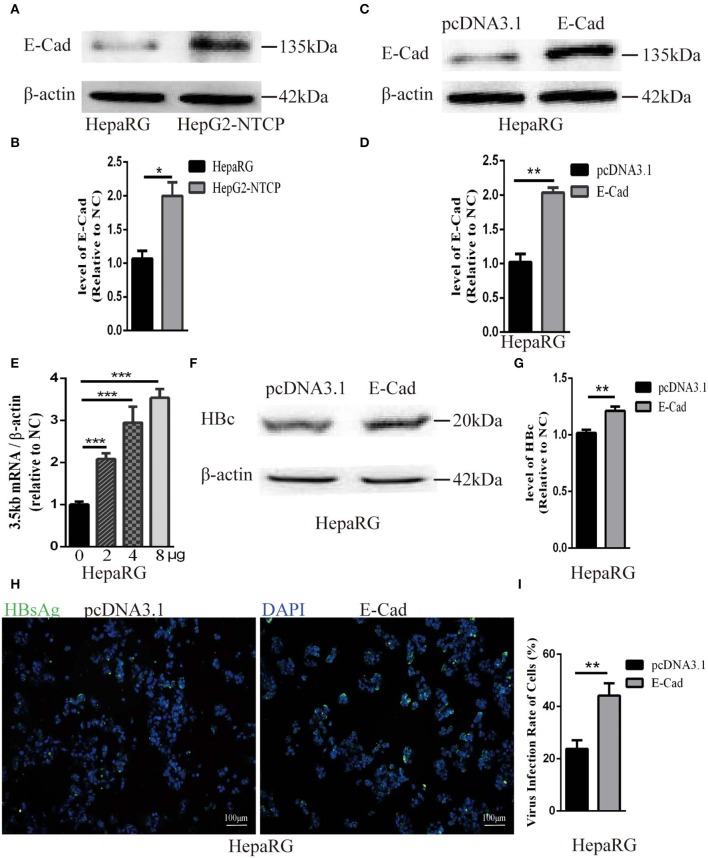
Overexpression of E-cadherin promotes HBV particles infection in HepaRG cells. **(A)** E-cadherin expression was assessed via western blot analysis in HepaRG and HepG2-NTCP cells. **(B)** The densitometric ratios were normalized to the β-actin and then compared to the controls. **(C)** Four microgram of pcDNA3.1-E-cadherin was transfected into HepaRG cells planted in 6-well plate. E-cadherin expression was assessed via western blot analysis 3 days post-transfection. **(D)** The densitometric ratios were normalized to the β-actin and then compared to the controls. **(E)** 0, 2, 4, and 8μg of pcDNA3.1-E-cadherin was transfected into HepaRG cells planted in 6-well plate. Then the cells were planted into 24-well plate at 2 days after transfection and then infected with HBV virus. Total RNA was extracted 2 days post-infection and the amount of HBV was assessed via qRT-PCR quantification of HBV 3.5 kb mRNA normalized to β-actin mRNA. **(F–I)** Four microgram of pcDNA3.1-E-cadherin was transfected into HepaRG cells planted in 6-well plate. Then the cells were planted into 24-well plate at 2 days after transfection and then infected with HBV virus. **(F)** Total protein was extracted 3 days post-infection and HBV core (HBc) expression was assessed by western blot analysis in HepaRG cells. **(G)** The densitometric ratios were normalized to the β-actin and then compared to the controls. **(H)** HBsAg expression was assessed by immunofluorescence 3 days post-infection in HepaRG cells. **(I)** The virus infection rate of the cells is the percentage of cells expressing green fluorescence calculated by the ImageJ-win 64. Representative data is shown from triplicate experiments. E-Cad, E-cadherin. *p < 0.05; **p < 0.01; ***p < 0.001; error bars: standard deviation (SD).

The concentration of E-cadherin was lower in HepaRG cells by about 50% compared to HepG2-NTCP cells (Figures 3A,B). Therefore, to elucidate the effect of E-cadherin overexpression on HBV infection, HepaRG cells were transfected with either pcDNA3.1-E-cadherin (E-cadherin) or pcDNA3.1 (vector). After transfected with pcDNA3.1-E-cadherin, the level of E-cadherin was upregulated double (Figures 3C,D). At 3 days post-transfection, HepaRG cells were infected with enriched HBV particles. E-cadherin overexpression increased the level of HBV 3.5 kb mRNA and in a concentration-dependent manner (Figure 3E). Furthermore, expression of recombinant E-cadherin enhanced the level of HBV core and HBsAg proteins (Figures 3F–I). These results suggest that E-cadherin contributed to HBV infection.

### E-cadherin Specifically Modulates HBV pseudoparticle Entry

**Figure 4:**
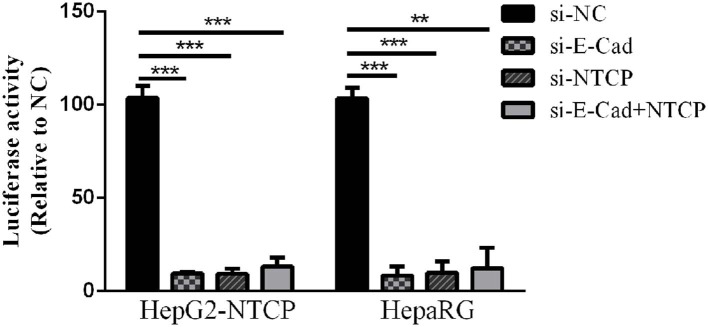
Downregulation of E-cadherin inhibits entry of HBV pseudoparticles. HepG2-NTCP and HepaRG cells were infected with luciferase-encoding pseudotyped virus particles bearing HBV large envelope glycoprotein LHBs 3 days post-transfection with siRNA. Luciferase assays were performed 72 h after infection. The value of si-NC group was normalized to 100 and the values of other groups were compared to the controls. Representative data is shown from triplicate experiments. **p < 0.01; ***p < 0.001; error bars: standard deviation (SD).

Lentivirus-based pseudoparticles had been used to study the entry pathways of a range of viruses, including human immunodeficiency virus (HIV) and hepatitis C virus (Moller-Tank and Maury, 2015). To further clarify the steps of the HBV life cycle impacted by E-cadherin expression, HBV pseudotyped virus (HBVpps) was conducted. HBVpps could only infect cells, but could not replicate within cells. Therefore, the amount of virus entering into the cell could be detected by measuring the luciferase activity. Silencing of E-cadherin or NTCP individually or together by siRNAs served to significantly inhibit HBVpps entry into HepG2-NTCP and HepaRG cells (Figure 4), suggesting that E-cadherin impacts HBV binding/entry.

### Silencing of E-cadherin Inhibits HBV Pre-S1 Binding and Internalization to HepG2-NTCP and PHH Cells

**Figure 5:**
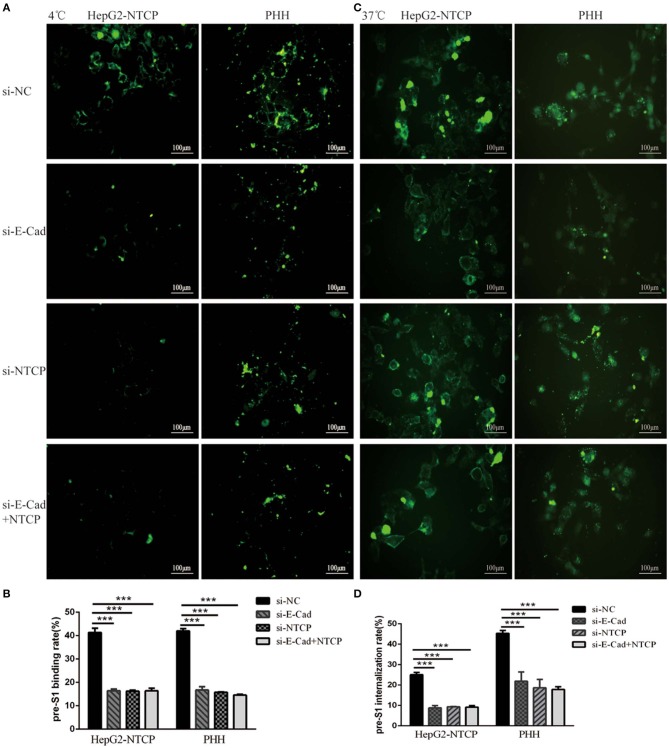
Silencing E-cadherin inhibits HBV preS1 binding and internalization by HepG2-NTCP and PHH cells. HepG2-NTCP and PHH cells were cultured with HBV preS1 for 2 h at **(A)** 4°C or **(C)** 37°C 3 days after transfection with si-NC, si-E-cadherin, si-NTCP or si-E-cadherin, and si-NTCP together. The binding or internalization rate was quantified by counting the total number of cells in the white light and the number of cells expressing fluorescence in the cell membrane or inside the cell. ***p < 0.001; error bars: standard deviation (SD) **(B,D)**. Representative data is shown from triplicate experiments.

We next sought to determine the mechanism employed by E-cadherin to enhance HBV binding. We, therefore, quantified the binding of HBV pre-S1 (Myr-2-47aa) via immunofluorescence assays in HepG2-NTCP and PHH cells after 120 min incubation at 4°C, which is the conditions in which viral binding most readily occurs, or at 37°C, when uptake of pre-S1 occurs. Results revealed that when E-cadherin or NTCP were silenced separately or at the same time, significant inhibition of preS1 binding and uptake were observed in HepG2-NTCP and PHH cells (Figure 5). These results suggest that E-cadherin modulates HBV entry by affecting preS1 binding and internalization by host cells.

### E-cadherin Regulates NTCP Cell Surface Distribution

**Figure 6:**
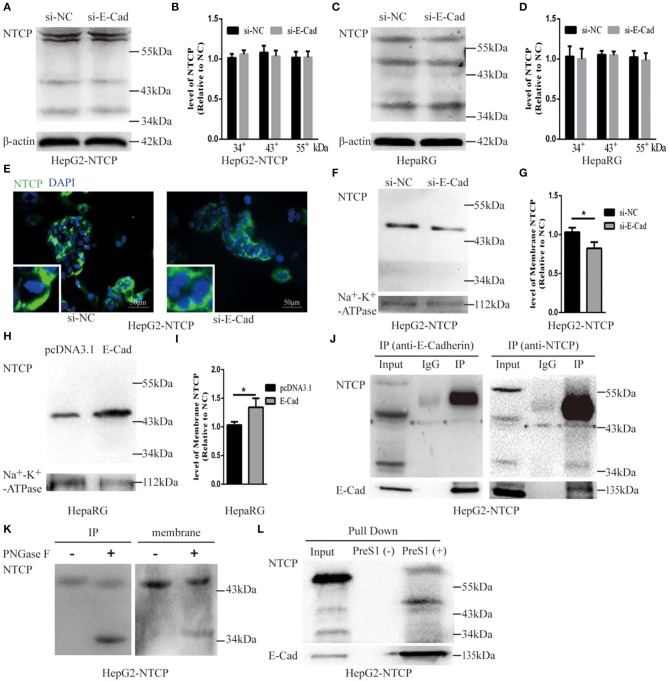
E-cadherin facilitates NTCP localization to the cell surface through interacting with glycosylated NTCP. Total protein was extracted 3 days post-transfection with siRNA-NC or siRNA-E-cadherin. NTCP expression was assessed by western blot analysis in **(A)** HepG2-NTCP and **(C)** HepaRG cells. The samples were derived from the same experiment and gels were processed in parallel. **(B,D)** The densitometric ratios of every band were normalized to the β-actin and then compared to the controls. There was no significant difference between the groups. **(E)** NTCP expression was assessed by immunofluorescence 3 days post-transfection with E-cadherin siRNA in HepG2-NTCP cells. **(F)** Membrane protein was extracted 3 days post-transfection with siRNA-NC (left panel) or siRNA-E-cadherin (right panel) and NTCP expression was assessed by western blot analysis in HepG2-NTCP cells. **(G)** The densitometric ratios were normalized to the Na^+^-K^+^-ATPase and then compared to the controls. **(H)** Membrane protein was extracted 4 days post-transfection with pcDNA3.1 plasmid or pcDNA3.1-E-cadherin and NTCP expression was assessed by western blot analysis in HepaRG cells. **(I)** The densitometric ratios were normalized to the Na^+^-K^+^-ATPase and then compared to the controls. **(J)** Co-immunoprecipitation was performed to confirm the interaction between E-cadherin and NTCP. Input: Total protein from cell extract. IgG of rabbit was used as the control group. IP: Total protein from Hep2-NTCP cells was incubated with anti-E-cadherin, or anti-NTCP at 4°C overnight. **(K)** The precipitate of IP and membrane protein were treated by PNGase F at 37°C for 1 h and detected by western blot. **(L)** The HepG2-NTCP cell lysates were incubated with pre-S1 to confirm that preS1 was bound to glycosylated NTCP. Representative data is shown from triplicate experiments. *p < 0.05; error bars: standard deviation (SD).

To further explore the mechanism by which E-cadherin mediates HBV particle entry, we examined whether it influenced the total NTCP concentration. The densitometric ratios of every bands were normalized to the β-actin and then compared to the controls. Results show that silencing of E-cadherin did not affect the level of NTCP in HepG2-NTCP and HepaRG cells (Figures 6A–D), which suggests that E-cadherin does not modulate HBV entry by directly affecting the expression and stability of NTCP. We, therefore, speculated that E-cadherin might influence the membrane distribution of NTCP. Our results showed that silencing E-cadherin in HepG2-NTCP cells significantly impacted the subcellular distribution of NTCP. Specifically, NTCP which primarily localizes to the cell surface was instead gathered in the cytoplasm (Figure 6E). We next separated membrane proteins to confirm the observed changes in NTCP cell surface distribution. Na^+^-K^+^-ATPase mainly distributed in the cell membrane was used as an internal reference. As shown in Figures 6F,G, knockdown of E-cadherin resulted in reduced levels of NTCP at the cell membrane in HepG2-NTCP cells. Besides, the data also indicated that overexpression of E-cadherin increased the levels of NTCP at the cell membrane in HepaRG cells (Figures 6H,I).

To elucidate the mechanism employed by E-cadherin to impact the cellular distribution of NTCP, we further examined the interactions between E-cadherin and NTCP via co-immunoprecipitation (co-IP). Surprisingly, when an E-cadherin antibody was used to precipitate the cellular lysate of HepG2-NTCP cells, an ~50 kDa protein band was detected in the precipitates with the NTCP antibody. To confirm whether the 50 kDa protein was glycosylated NTCP, the precipitate was treated by PNGase F and further probed with NTCP antibodies. Results demonstrated that PNGase F treatment caused the 50 kDa protein to change into a 39 kDa variant; suggesting the 50 kDa protein was a glycosylated form of NTCP (Figure 6J). Furthermore, after treating cell membrane proteins with PNGase F, we also observed a shift in electrophoresis size from 50 to 39 kDa, indicating that a large fraction of the NTCP localized at the cell membrane was glycosylated (Figure 6K). Lastly, after incubating preS1 with HepG2-NTCP cell lysates, we observed the formation of preS1, glycosylated NTCP and E-cadherin complexes (Figure 6L). Taken together these results suggest that E-cadherin binds to glycosylated NTCP, allowing for efficient localization to the cell surface.

## Discussion

HBV entry into the host cell is the critical step in their life cycle. This process includes particle delivery and capture, complex internalization, and membrane fusion (Hayes et al., 2016). HBV entry requires a tightly coordinated group of specific viral proteins and multiple host receptors (Miao et al., 2017). In the current study, we report that E-cadherin acts as a novel host factor that facilitates HBV entry. The functional role of E-cadherin as a host entry factor was confirmed by numerous studies. Specifically we showed that silencing of E-cadherin acted to inhibit HBV particle entry into HepG2-NTCP, HepaRG and PHH cells (Figures 1, 2); overexpression of E-cadherin contributed to HBV particle entry in HepaRG cells (Figure 3) and silencing of E-cadherin inhibited HBVpps entry in HepG2-NTCP and HepaRG cells (Figure 4). Moreover, E-cadherin silencing caused a significant decrease in the binding and internalization of the HBV pre-S1 peptide by HepG2-NTCP and PHH cells (Figure 5). Mechanistic studies suggested that E-cadherin regulates the cell-surface distribution of NTCP (Figure 6). This study represents an important step forward in understanding the molecular mechanisms and cellular regulatory events involved in HBV entry.

Although Li J. et al. (2016) reported that HepG2-NTCP cells exhibited poor susceptibility to HBV particles derived from patient serum, our study demonstrated the opposite effect with HepG2-NTCP cells being efficiently infected with HBV particles from serum of chronic HBV patients. These results were confirmed by quantifying specific markers of HBV infection (HBV 3.5 kb RNA, HBc and HBsAg) to confirm infection of HepG2-NTCP cells (Figure 2). Similarly, Yan et al. (2012) successfully infected HepG2-NTCP cells with an HBV genotype B virus from the plasma of a carrier with HBV. An additional study also reported successful infection of HepG2-NTCP cells with an HBV genotype D virus isolated from serum to determine the effect of glypican-5 (GPC5) expression on HBV entry (Verrier et al., 2016).

E-cadherin is a type I classical cadherin and a calcium-dependent adhesion glycoprotein expressed in the epithelium (Harris and Tepass, 2010). E-cadherin has been described as a critical component in the regulation of pathways highly associated with cancer development, including cellular proliferation, apoptosis, invasiveness, metabolism, and metastasis, through mediating multiple cellular signaling pathways (Yulis et al., 2018). Many additional studies have focused on the effect that E-cadherin has on pathogenic infections caused by bacteria and viruses (Bonazzi and Cossart, 2011). *Listeria monocytogenes* is a foodborne pathogen that crosses the intestinal barrier upon binding between its surface protein InlA and the host receptor E-cadherin occurs (Nikitas et al., 2011). *Candida albicans* produces two types of invasins, namely, Als1 and Als3, in combination with E-cadherin or N-cadherin, which together facilitate the internalization of bacteria (Phan et al., 2007). Moreover, E-cadherin associates directly with nectin-1 which is critical for HSV-1 infection (Drees et al., 2005). Connolly et al. (2005) also reported that E-cadherin overexpression may strengthen intercellular junctions and shorten the distance between infected and uninfected cells, thereby increasing the regional concentration of nectin-1 to facilitate efficient viral spread. E-cadherin was also found to play a critical role in HCV entry through regulating membrane distribution of the HCV receptors, claudin-1 (CLDN1) and occludin (OCLN) (Li Q. et al., 2016). These studies provide evidence that E-cadherin acts directly as a receptor while also modulating the expression and activity of other receptors to mediate pathogenic infections.

Herein we show that E-cadherin plays a critical role in HBV entry by influencing the distribution of NTCP, the functional receptor of HBV. NTCP, a 349 residue glycoprotein, becomes glycosylated at N5 and N11 in the endoplasmic reticulum and Golgi apparatus before being trafficked to the cell surface, resulting in a band that spans from 39 to 56 kDa (Appelman et al., 2017). Surprisingly, we found that E-cadherin interacts with the glycosylated form of NTCP (Figure 6E). Therefore, when E-cadherin is distributed on the cell membrane, the glycated NTCP bound to E-cadherin could also be localized to the cell membrane. A previous study indicated that glycosylation is essential for NTCP to act as a receptor for HBV since the non-glycosylated form of NTCP is rapidly internalized and degraded (Appelman et al., 2017). Yan et al. (2012) also found that Myr-preS1 cross-links with glycosylated NTCP. However, another study claimed that both the glycosylated and non-glycosylated forms of NTCP effectively mediate HBV infection, since differentiated HepaRG cells only express non-glycosylated NTCP (Lee et al., 2018). In our study, both glycosylated and non-glycosylated NTCP were detected in differentiated HepaRG and HepG2-NTCP cells, however, only glycosylated NTCP were detected in membrane proteins. Moreover, Myr-2-47aa was found to bind to the glycosylated form (Figure 6G). Our results showed that E-cadherin was associated with glycosylated NTCP (Figure 6E), and, thus, we propose that E-cadherin exerts a regulatory role on the cellular entry of HBV through interacting with glycosylated NTCP and facilitating its membrane localization in hepatocytes. Moreover, NTCP is a hepatic Na^+^ bile acid symporter and is responsible for cotransportation of sodium and bile acids across cellular membranes to maintain the enterohepatic circulation of bile acids (Stieger, 2011). Whether E-cadherin could affect the physiological roles of NTCP such as transportation of sodium and bile acids need to be further studied.

Furthermore, E-cadherin is the primary component of adherens junctions at the basolateral surfaces of polarized epithelial cells and function to establish cellular polarity (Shneider et al., 1997). Cell polarization, defined as the asymmetric distribution of components and functions in a cell, is not only required for proper cellular functioning but has also been defined as being associated with pathogenic infection (Ruch and Engel, 2017). The archetypal polarized animal cell is the epithelial cell, however, hepatocytes are also highly polarized. Pathogens such as *Helicobacter pylori* (Tammer et al., 2007), *Salmonella typhimurium* (Liao et al., 2008), *Shigella dysenteriae* (Beau et al., 2007), and Rotavirus (Guglielmi et al., 2007), either interfere with the establishment of cellular polarization, or adapt to use polarized molecules as their functional receptors, thereby facilitating self-infection of host cells. Furthermore, Schulze et al. (2012) reported that hepatocyte polarization is essential for HBV entry. So, E-cadherin may affect HBV entry through affecting hepatocyte polarization and this need to be studied in the future.

In summary, E-cadherin improved the rate of HBV entry through binding to glycosylated NTCP thereby, impacting the membrane distribution of NTCP. This study provides novel insights that advance the current understanding of the HBV life cycle as well as inform the development of pharmaceutical interventions targeting E-cadherin as a means to prevent HBV infection.

## Data Availability Statement

All datasets generated for this study are included in the article/supplementary material.

## Ethics Statement

The studies involving human participants were reviewed and approved by Ethics Committee of the Second Affiliated Hospital of Chongqing Medical University. The patients/participants provided their written informed consent to participate in this study.

## Author's Note

The following reagent was obtained through the NIH AIDS Reagent Program, Division of AIDS, NIAID, NIH: pNL4-3.Luc.R–.E– from Dr. Nathaniel Landau. This manuscript has been released as a Pre-Print at https://www.biorxiv.org/content/10.1101/729822v1 (Hu et al., 2019).

## Author Contributions

WC designed the study. QH, FZ, LD, and BW conduct the experiments. QH, FZ, PL, YY, DL, and SY conducted analyses. QH wrote the manuscript. WC and LZ edited the manuscript.

### Conflict of Interest

The authors declare that the research was conducted in the absence of any commercial or financial relationships that could be construed as a potential conflict of interest.
